# Clonal expansion and rapid characterization of *Klebsiella pneumoniae* ST1788, an otherwise uncommon strain spreading in Wales, UK

**DOI:** 10.1099/mgen.0.001104

**Published:** 2023-09-05

**Authors:** Massimo Mentasti, Sophia David, Jane Turton, Mari Morgan, Luke Turner, Joseph Westlake, Jonathan Jenkins, Catie Williams, Sara Rey, Joanne Watkins, Victoria Daniel, Shanine Mitchell, Gavin Forbes, Mandy Wootton, Lim Jones

**Affiliations:** ^1^​ Specialist Antimicrobial Chemotherapy Unit, Public Health Wales Microbiology, Cardiff, CF14 4XW, UK; ^2^​ Centre for Genomic Pathogen Surveillance, Big Data Institute, University of Oxford, Oxford, OX3 7LF, UK; ^3^​ HCAI, Fungal, AMR, AMU & Sepsis Division, UK Health Security Agency, London, NW9 5HT, UK; ^4^​ Healthcare Associated Infection, Antimicrobial Resistance Prescribing Programme, Public Health Wales Health Protection, Cardiff, CF10 4BZ, UK; ^5^​ Bacteriology Department, Public Health Wales Microbiology, Swansea, SA2 8QA, UK; ^6^​ Pathogen Genomics Unit, Public Health Wales Microbiology, Cardiff, CF14 4XW, UK; ^7^​ Bacteriology Department, Public Health Wales Microbiology, Cardiff, CF14 4XW, UK

**Keywords:** ESBL, KL2, *Klebsiella pneumoniae*, multidrug resistance, PCR, ST1788, yersiniabactin

## Abstract

A multidrug-resistant strain of *Klebsiella pneumoniae* (*Kp*) sequence type (ST) 1788, an otherwise uncommon ST worldwide, was isolated from 65 patients at 11 hospitals and 11 general practices across South and West Wales, UK, between February 2019 and November 2021. A collection of 97 *Kp* ST1788 isolates (including 94 from Wales) was analysed to investigate the diversity and spread across Wales and to identify molecular marker(s) to aid development of a strain-specific real-time PCR. Whole genome sequencing (WGS) was performed with Illumina technology and the data were used to perform phylogenetic analyses. Pan-genome analysis of further *Kp* genome collections was used to identify an ST1788-specific gene target; a real-time PCR was then validated against a panel of 314 strains and 218 broth-enriched screening samples. Low genomic diversity was demonstrated amongst the 94 isolates from Wales. Evidence of spread within and across healthcare facilities was found. A yersiniabactin locus and the KL2 capsular locus were identified in 85/94 (90.4 %) and 94/94 (100 %) genomes respectively; *bla*_SHV-232_, *bla*_TEM-1_, *bla*_CTX-M-15_ and *bla*_OXA-1_ were simultaneously carried by 86/94 (91.5 %) isolates; 4/94 (4.3 %) isolates also carried *bla*_OXA-48_ carbapenemase. Aminoglycoside and fluoroquinolone resistance markers were found in 94/94 (100 %) and 86/94 (91.5 %) isolates respectively. The ST1788-specific real-time PCR was 100 % sensitive and specific. Our analyses demonstrated recent clonal expansion and spread of *Kp* ST1788 in the community and across healthcare facilities in South and West Wales with isolates carrying well-defined antimicrobial resistance and virulence markers. An ST1788-specific marker was also identified, enabling rapid and reliable preliminary characterization of isolates by real-time PCR. This study confirms the utility of WGS in investigating novel strains and in aiding proactive implementation of molecular tools to assist infection control specialists.

## Data Summary

Genome data generated and/or analysed in this work are available in the European Nucleotide Archive (ENA) under project number ERP135832 or PRJEB48990. Individual accession numbers are listed in Table S1, available in the online version of this article, or in the Methods.

**Impact Statement**

Multidrug-resistant (MDR) *Klebsiella pneumoniae* strains are an established cause of healthcare-associated infections, while hyper-virulent strains have been shown to cause severe infections in the community. Certain clones have been identified as ‘high risk’ and are more likely to cause significant disease and dissemination in healthcare settings. A previous study in Wales identified a high diversity of *K. pneumoniae* sequence types (STs) including the high-risk clones ST15, ST20 and ST307. Surprisingly, *K. pneumoniae* ST1788 was the second most common ST in Wales despite only few ST1788 genomes being found in public databases. ST1788 isolates were therefore investigated in this study to characterize their genomic diversity, resistance and virulence profiles, and their spread across Wales. A strain-specific rapid assay was designed and validated to allow prompt implementation of infection control measures. This study introduces *K. pneumoniae* ST1788 to the scientific community highlighting its MDR nature and the ability to spread within and outside healthcare settings. It also provides a proof of principle for the design and implementation of a rapid, cost-effective screening method to identify bacterial strains of clinical and public health concern, following characterization of new strains of interest by whole genome sequencing, as part of local or national surveillance programmes.

## Introduction

*Klebsiella pneumoniae* is a Gram-negative bacterium belonging to the order *Enterobacterales *, usually found as a commensal in the normal intestinal flora. It causes opportunistic infections in humans and is recognized as a leading cause of healthcare-acquired infections worldwide [1–3]. Clinical manifestations include pneumonia, skin and wound infections, urinary tract infections and sepsis, particularly in the elderly, neonates and immunocompromised individuals [4]. Direct consequences are increased hospital stays, treatment costs and mortality rates [5]. *Klebsiella pneumoniae* isolates are intrinsically resistant to penicillins due to the constitutive presence of *bla*_SHV_; they can also acquire a wide range of antimicrobial resistance (AMR) markers via horizontal gene transfer, including those encoding extended spectrum β-lactamases (ESBLs) and carbapenemases [6]. In 2017 the World Health Organisation (WHO) listed ESBL- and carbapenemase-producing *Enterobacterales *, including *K. pneumoniae *, amongst the ‘Priority 1: CRITICAL’ bacteria for which new antibiotics are urgently needed [7]. New β-lactamase inhibitors, such as avibactam, relebactam and vaborbactam, have since become available and are used in combination with pre-existing antibiotics, respectively ceftazidime, imipenem and meropenem, although they still lack efficacy against strains carrying *bla*_IMP_, *bla*_NDM_ and *bla*_VIM_ metallo-β-lactamases [8]. Cefiderocol, a siderophore cephalosporin, has shown stability against a wider range of β-lactamases, but there is currently a relative lack of data regarding clinical efficacy and issues with susceptibility testing [9–11].

In addition to AMR determinants*, K. pneumoniae* can carry virulence factors facilitating infection and worsening patient outcomes. A polysaccharide capsule protects from phagocytosis and the bactericidal activity of the complement system [12]. At least 134 *K *. *pneumoniae* capsular antigens (K) have been described, with K1 and K2 types being the most prevalent in highly virulent strains and usually associated with poorer outcomes [13–15]. Hyper-virulent K1 strains are generally associated with the sequence type (ST) 23 lineage and are usually isolated in Asian countries, while K2 strains are more geographically widespread [13]. The ability to hyper-produce capsular polysaccharide components, mediated by the presence of *rmpA* and *rmpA2*, is recognized as a further increase in virulence capacity [16]. Further virulence factors include the siderophores aerobactin, salmochelin and yersiniabactin, which are responsible for scavenging iron, a crucial nutrient, from host transport proteins, facilitating *K. pneumoniae* survival and replication within the host [17–19]. A further siderophore known as enterobactin is ubiquitous in *K. pneumoniae* strains but it is inactivated by the lipocalin 2 protein in the host and therefore is unlikely to play a major role during pathogenesis [20]. Siderophores are usually carried on mobile genetic elements that can also carry additional virulence determinants, such as colibactins which are able to induce dsDNA breaks in eukaryotic cells [21].

Specific *K. pneumoniae* lineages such as ST15, ST101, ST147, ST258, ST307 and closely related strains, especially those carrying ESBLs and carbapenemases, have been shown to be very effective at spreading within and between healthcare facilities worldwide [22]. A survey of European hospitals in 2013–14 showed that 69.9 % (477/682) of carbapenemase-producers were concentrated in four clonal lineages, namely ST11, ST15, ST101 and ST258/512, plus closely related STs that evolved from them. Furthermore, the ability to spread in hospital environments was correlated with the degree of AMR, with carbapenemase producers showing the highest degree of transmissibility [23].

The Specialist Antimicrobial Chemotherapy Unit (SACU) based at the University Hospital of Wales in Cardiff, UK, provides an antimicrobial reference service for aerobic bacteria, including *K. pneumoniae *, that are referred by microbiology laboratories across Wales. In parallel, SACU collects isolates from blood cultures for surveillance purposes and receives isolates from suspected outbreaks as part of the development of a whole genome sequencing (WGS)-based typing programme. A study describing the WGS analysis of more than 500 *Klebsiella* strains isolated in Wales identified *K. pneumoniae* ST1788 as the second most common *K. pneumoniae* strain referred to SACU; the most common was ST307, a sequence type associated with at least one known outbreak in Wales [24]. ST1788 has been reported only rarely in the literature, including reports of single isolates from Gabon, Ghana and Nigeria [25–27].

By contrast, 43 ST1788 isolates were referred to SACU between February 2019 and November 2020 from specimen types ranging from screening samples to blood cultures. Almost all strains were ESBL producers, resistant to ceftazidime and cefotaxime, and also resistant to gentamicin and to ciprofloxacin; three isolates from a single patient also carried *bla*_OXA-48_ carbapenemase and were resistant to ertapenem, while variably resistant to imipenem and meropenem.

To better monitor spread of this strain, a routine screening programme for isolation of ESBL-producing *K. pneumoniae* was implemented in wards of two hospitals (B and C). Concurrently, an enhanced laboratory protocol using an in-house selective broth was introduced to increase overall sensitivity of the screening programme; performance of the two screening methods was compared over a period of 20 weeks. Finally, genomic approaches were used to characterize the diversity and spread of ST1788 in Wales; an ST1788-specific target was identified to develop a strain-specific real-time PCR to rapidly provide preliminary typing data and so assist implementation of relevant infection control measures.

## Methods

### Epidemiological analysis

Patient demographics and hospitalization histories were extracted from ICNet (Baxter), an infection prevention case management system used in all Welsh hospitals, using identifiers provided for SACU laboratory submission.

### Bacterial strains

A total of 314 previously characterized strains from 25 different species were selected from the American Type Culture Collection (ATCC), the National Collection of Type Cultures (NCTC) and the authors’ collection (Tables S1 and S2). The panel included 275 previously sequenced *Klebsiella* spp. isolates chosen to represent the wide variety of species and *K. pneumoniae* lineages previously identified in Wales [24]. Included amongst them were 91 *K *. *pneumoniae* ST1788 strains from 65 patients plus three environmental strains isolated between February 2019 and November 2021; 43/94 isolates had already been characterized in the above-mentioned study and a further 51 were isolated and included in this study. In total, 47/94 were isolated from screening samples (19 urine samples, 17 rectal swabs, 11 stools), 44/94 from clinical samples (25 urine samples, 14 blood cultures, two tissue samples, one cerebrospinal fluid, one wound swab, one pus swab) and 3/94 from environmental screening swabs.

Bacterial strains were stored on cryogenic beads at −80 °C and aerobically grown overnight at 35±1 °C on Columbia Blood Agar (CBA; Oxoid, Thermo Fisher) before analysis.

### Whole genome sequencing

DNA was extracted from the 51 new *K. pneumoniae* ST1788 isolates, and Nextera libraries were prepared and then sequenced on MiSeq or NextSeq (Illumina) platforms as previously described [24]. Short read data were made available on the European Nucleotide Archive (ENA) under project accession ERP135832; short read data for the 43 ST1788 strains previously sequenced are also available on the ENA under project accession PRJEB48990. Individual accession numbers for the sequenced isolates are detailed in Table S1.

### Characterization of ST1788 genomes and phylogenetic analysis

WGS data from 94 ST1788 isolates from Wales (Table S1), together with three ST1788 genomes from Nigeria (SRR5513537, SRR5513549 and ERR4783559) identified via the Pathogenwatch database [28], were analysed in this study. Short sequence reads were assembled using SPAdes v3.10.0 [29] using the ‘--careful’ flag and the ‘--cov-cutoff’ flag set to ‘auto’. Assemblies were annotated using Prokka v1.14.5 [30]. Kleborate v2.0 [31] was used to determine assembly quality metrics, confirm the species and ST from each assembly, identify virulence and resistance genes, and determine the capsule and O antigen biosynthesis loci via Kaptive [14]. Plasmid replicons were identified via Pathogenwatch [27] using the PlasmidFinder database [32]. The presence of a pOXA-48-like plasmid was inferred by mapping short reads of isolates to the pOXA48a reference sequence (accession JN626286) using Burrows Wheeler Aligner [33], SAMtools v1.2 mpileup and BCFtools v1.2 [34]. The resulting BAM file was then used to determine the length of the plasmid that was mapped by at least one sequence read.

To generate a phylogenetic tree of ST1788 isolates, sequence reads were mapped to the complete chromosomal sequence from a hybrid assembly of an ST1788 isolate generated previously (ARGID_33542; accession GCA_922827155) [24]. Mapping was performed using Burrows Wheeler Aligner [33] with SNPs identified using SAMtools v1.2 mpileup and BCFtools v1.2 [34]. Gubbins v2.4.1 [35] was used to remove recombined regions from the resulting pseudo-genome alignment and generate a maximum-likelihood phylogeny based on the remaining variable positions. The phylogeny was visualized together with available metadata and genotyping data using Microreact [36]. Pairwise SNP differences were calculated using the pseudo-genome alignment after the removal of recombined regions.

### Identification and validation of targets for the ST1788-specific PCR assay

Candidate genes for an ST1788-specific PCR assay were initially identified using Panaroo v1.2.4 in the ‘moderate’ mode [37]. This was run on a diverse public collection of 1717 genomes from the *K. pneumoniae* species complex [23] together with a single representative ST1788 genome (ARGID_35371) from Wales. The presence of the resulting candidate genes in additional genomes was further determined using BLASTn [38] with a database including all ST1788 genomes in our collection (*n*=94), additional *Klebsiella* genomes from Wales sequenced previously (*n*=497) [24] and a large public genome collection of *K. pneumoniae* genomes from Pathogenwatch (*n*=16086) [28] . The genes were also queried against the NCBI-nr database using BLASTn.

### Oligonucleotide design and analysis

Primers and probes were designed using Primer-blast [39] following criteria present on the Rotor-Gene Multiplex PCR Kit manual (Qiagen) to optimize multiplex real-time PCR assays. An *in silico* PCR [40], allowing for a maximum of three mismatches in each of the primer binding sites to define a positive result, was then performed to initially identify possible cross-reactions against the above mentioned public collection of 1717 *K *. *pneumoniae* species complex genomes [23].

Oligonucleotide sequences and amplicon sizes are detailed in Table 1; the nucleotide sequence of the targets with highlighted PCR fragments is available in the Supplementary Information.

**Table 1.** Summary of oligonucleotide sets used in this study (FP, forward primer; RP, reverse primer; HP, hydrolysis probe)

Target		Name	Sequence (5′→3′)	Final concentration (nM)	Amplicon size (bp)	Reference
ST1788	04677	04677_FP	GAACTAGCGGTGGTCAGGAA	250	126	This study
		04677_RP	CAAGTGACAATAGCGCAACGTC	250		
		04677_HP	FAM-TGGCGTGATCAGGCTGTTAGTATTGC-BHQ1	100		
* Klebsiella pneumoniae *	*yphG*	yphG_FP	GAGTTAGGGAAACGAACATTGTG	250	169	[43]
		yphG_RP	TCTCTATCGGACAGACGTCG	250		
		yphG_HP	HEX-TTCATTGGCATCATCACTTAGCGAC-BHQ1	100		
Internal Process Control	*gfp*	gfp _FP	CCTGTCCTTTTACCAGACAACCA	100	76	[44]
		gfp _RP	GGTCTCTCTTTTCGTTGGGATCT	100		
		gfp_HP	DY682-TACCTGTCCACACAATCTGCCCTTTCG-BBQ650	100		

### *In vitro* analysis

#### Antimicrobial susceptibility testing

All 94 *K *. *pneumoniae* ST1788 isolates were tested against 12 antibiotics (ampicillin, amoxicillin/clavulanate, piperacillin/tazobactam, ceftazidime, cefotaxime, ertapenem, imipenem, meropenem, amikacin, gentamicin, ciprofloxacin and trimethoprim/sulfamethoxazole) following EUCAST guidelines for disc diffusion methodology on Mueller Hinton agar (Oxoid, ThermoFisher) and for result interpretation [41, 42]. Where minimum inhibitory concentration (MIC) results were required, or interpretation was unclear or not possible (e.g. inhibition zone within area of technical uncertainty), the antibiotic was retested using a gradient strip (bioMérieux or Liofilchem) and results again interpreted following EUCAST guidelines [42].

#### Screening samples

A standard screening programme was implemented over a 20 week period (late July to mid-December 2021) at Hospital B and Hospital C; samples were collected at each patient’s admission and then on a weekly basis. A total of 527 samples (194 urine samples, 186 rectal swabs and 147 stools) were collected from 89 patients (one to 36 samples per patient) and submitted for isolation of ESBL-producing *K. pneumoniae* (Table S3). Briefly, on the day of receipt samples were plated onto Brilliance ESBL Agar (Oxoid, Thermo Fisher) and after 24/48 h of incubation at 35±1 °C, identification of putative *K. pneumoniae* colonies was confirmed by MALDI-TOF (Bruker) before isolates were referred for PCR detection of ST1788 and WGS analysis. Screening samples were then stored at 2–8 °C and on the following Monday (i.e. 3–7 days later) they were processed using an enhanced screening protocol. Briefly, samples were inoculated onto 10 ml of Mueller Hinton Broth (MHB; Oxoid, ThermoFisher) containing ceftazidime (4 mg l^−1^), cefotaxime (8 mg l^−1^), gentamicin (8 mg l^−1^) and vancomycin (8 mg l^−1^). Rectal swabs were directly inoculated into the broth while urine samples were mixed by vortexing before inoculating 100 µl; pea-size stool aliquots were vigorously resuspended by vortexing into 3 ml of saline (Oxoid, ThermoFisher) and then 100 µl of this solution was used as inoculum. In parallel, 100 µl of a 0.5 McFarland dilution of *K. pneumoniae* SACU_35765 (ST1788) and *Escherichia coli* ATCC 25922 were respectively used as positive and negative broth controls. After 24 h of incubation, broths with visible growth were sub-cultured onto Brilliance ESBL Agar (Oxoid, ThermoFisher) and CLED Agar (Oxoid, ThermoFisher); clear broths were incubated for a further 24 h before being sub-cultured or discarded. Positive broths were processed by PCR for detection of ST1788 markers and putative *K. pneumoniae* colonies were again confirmed by MALDI-TOF (Bruker) and submitted for WGS.

#### ST1788 detection by real-time PCR

A real-time PCR was optimized for the Rotor-Gene Q 5PLEX_HRM_ (Qiagen) to detect *yphG,* a previously published *K. pneumoniae *-specific target, using oligonucleotides modified from a previously published assay [43] and an ST1788-specific target identified in this study; a further assay detecting the green fluorescent protein gene*, gfp*, from *Aequorea victoria* [44] was also included as an Internal Process Control (IPC) to discount PCR inhibition. After standard screening, bacterial growth was harvested from agar media using a 10 µl loop (approximately a third full), resuspended by vortexing into 1 ml of PCR-grade water and pelleted by centrifugation at 12000 ***g*** for 2 min; while for enhanced screening, growth was harvested from 500 µl of positive MHBs by centrifugation at 12000 ***g*** for 2 min; after removal of supernatant, DNA was extracted from pellets as previously described [45]. A positive control (* K. pneumoniae* ST1788 SACU_35765) and an extraction control (nuclease-free water for isolate extraction and sterile MHB for broth extractions) were included in every extraction. A custom-made plasmid (Eurofins) containing the entire *gfp* sequence (Accession: M62653) was diluted to a working concentration of 1 pmol µl^–1^ before being added to the reaction mix. Reactions were performed in a final volume of 20 µl containing 5 µl of QuantiNova Multiplex PCR Kit (Qiagen), 11.5 µl of PCR-grade water, 1 µl of 20× oligonucleotide mix, 0.5 µl of pGFP (1 pmol µl^–1^) and 2 µl of template. The final concentration of each oligonucleotide is listed in Table 1. The *Taq* polymerase was activated at 95 °C for 2 min, followed by 40 cycles of denaturation at 95 °C for 5 s and annealing/extension at 60 °C for 30 s. Amplification results were analysed ignoring fluorescence in the first 10 cycles; a threshold of 0.05 was set for the Yellow (*yphG*) and Crimson (IPC) channels, while 0.1 was used for the Green (ST1788) channel of the Rotor-Gene Q 5PLEX_HRM_ (Qiagen).

#### Variable number tandem repeatanalysis and capsular typing

Variable number tandem repeat (VNTR) analysis, and PCR detection of K1, K2, K5, K20, K54 and K57 capsular types and *rmpA*/*rmpA2* capsule up-regulation genes were carried out as previously described [46].

## Results

### Epidemiological analysis

In total, 55 % (36/65) of the Wales ST1788 patients were male with ages ranging from 17 to 96 years (median=66 years). Eighty-three per cent (54/65) of patients had first samples submitted from 11 hospitals across South and West Wales; the number of patient cases per hospital ranged from one to 26. The remaining 17 % (11/65) of patients had first samples submitted from 11 general practices; most had histories of prior hospitalization, but only three immediately prior to specimen collection (Table S1).

### *In silico* analysis

#### Phylogeography of ST1788 in Wales

**Fig. 1.**
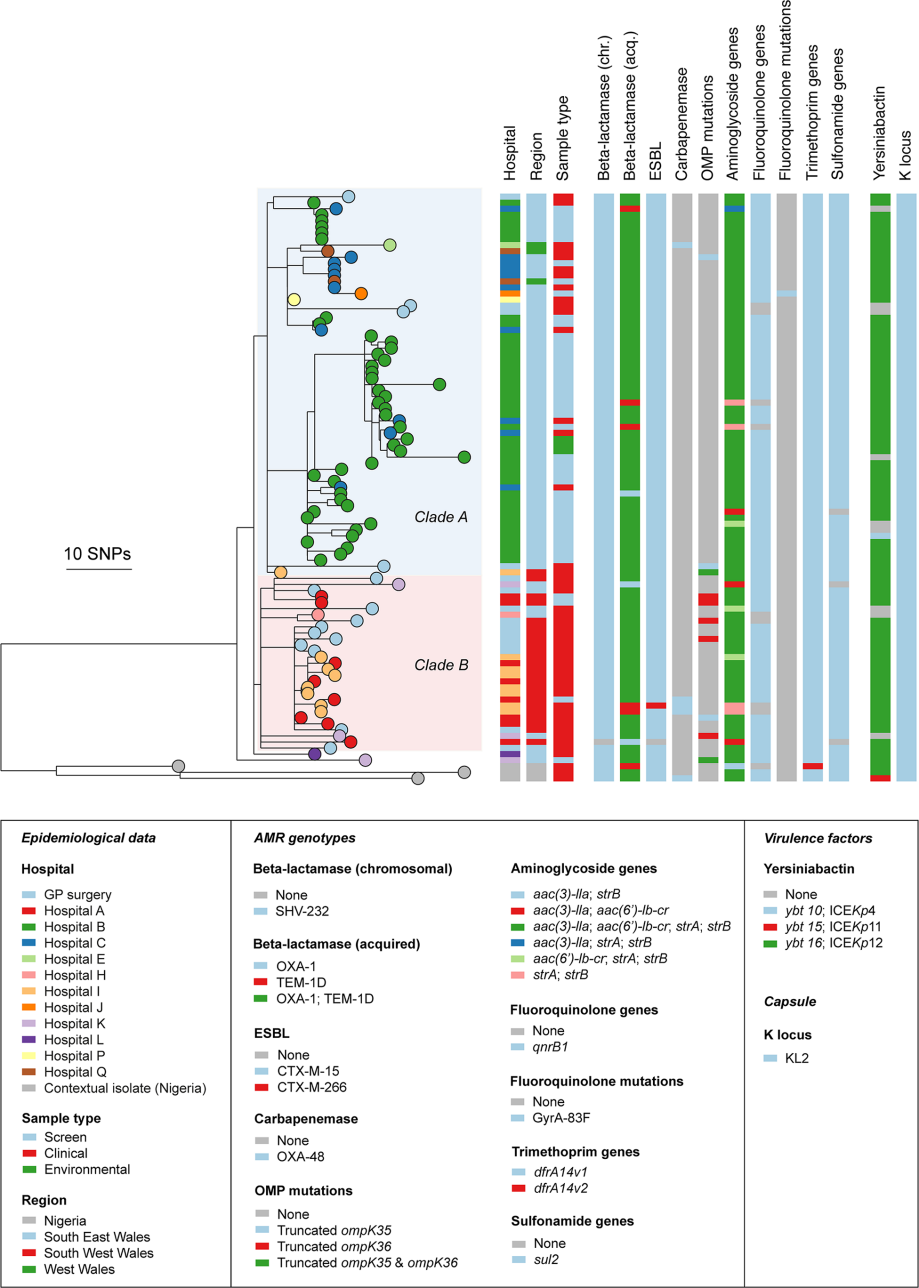
Maximum-likelihood phylogenetic tree of 97 ST1788s shows limited genomic diversity among isolates from Wales. The tree includes 94 isolates from Wales and three from a public genome collection (all from Nigeria). It was constructed from a pseudo-genome alignment generated by mapping short sequence reads to an ST1788 reference (ARGID_33542) and after the removal of recombined regions. Metadata columns show the sampling hospital, sample type, presence of mutations and genes linked to antibiotic resistance, virulence genes and the K locus. The scale bar represents the number of SNPs. The tree is available to view interactively and with additional metadata at https://microreact.org/project/kp-st1788-phw.

Phylogenetic analysis showed that the 94 *K *. *pneumoniae* ST1788 genomes from Wales clustered together, with pairwise SNP differences ranging from 0 to 48 (Fig. 1); 92/94 (97.9 %) of these formed two separate monophyletic clades (A and B) (Fig. 1) which are discussed in detail below. The additional three genomes from Nigeria were positioned outside of the Welsh clade and had a minimum of 60 (SRR5513537), 85 (SRR5513549) and 94 (ERR4783559) SNP differences with isolates from Wales (Table S5). ST1788 diversity amongst the 18 patients from whom multiple isolates were collected (range: two to four isolates per patient) was also assessed. Overall, low diversity was found with a maximum of five SNPs among 12 sets of same-patient isolates and a maximum of 15 SNPs among the remaining six sets.

The larger clade (A) in the upper part of the ST1788 tree comprises 60 isolates from 41 patients obtained from seven hospitals and three general practices, plus three environmental isolates from Hospital B. The majority of isolates in this clade (53/63; 84.1 %) were collected from Hospital B and Hospital C in South East Wales which are administratively linked and have frequent inter-hospital transfers. Forty-three isolates from 26 patients were part of an outbreak at Hospital B which was defined on the basis of the identical ST and corresponding VNTR profile (where available), plus admission to a specific unit within Hospital B. The three environmental isolates collected from Hospital B, clustered together in the tree and had two to four SNP differences (Table S5) with the closest clinical isolate in clade A (Fig. 1). Ten isolates from eight patients were collected from Hospital C, including three invasive isolates (SACU_35765, SACU_36212 and SACU_36213) which clustered very closely to isolates from different patients in Hospital B (one to four SNP differences; Table S5). This finding supports multiple instances of strain spread between the two hospitals. A further three strains from three patients in this clade were collected at Hospital J or Hospital P in South East Wales or Hospital Q in West Wales, but were either repeat specimens from patients previously admitted to Hospital C or new specimens from patients recently discharged from Hospital C. Finally, three strains from three patients in Hospital E and Hospital I in South West Wales and Hospital Q in West Wales, and four strains in three patients from General Practices in South East Wales, had no known recent history of admission in Hospital B or Hospital C.

Clade B in the lower part of the tree includes 29 strains from 22 patients. Fifteen strains from 11 patients were isolated at Hospital A or Hospital I in South West Wales, which are administratively linked; one patient (n. 173) in particular had a history of admission and strains (SACU_33405, SACU_33476 and SACU_33542) isolated at both hospitals. In this clade also there is evidence of wider geographical spread of strains as it includes three isolates from three patients admitted to Hospital H or Hospital K located in South East Wales, with no admission history to hospitals A or I. Finally, interspersed within this clade are 11 strains isolated from eight patients attending General Practices located both in South East and South West Wales; four of these strains were from four patients with no history of admission to Hospital A or Hospital I.

## AMR markers

During this study a new *bla*_SHV_ variant and a new *bla*_CTX-M_ variant were identified; these variants were submitted to GenBank and later designated as *bla*_SHV-232_ (Accession OR224963) and *bla*_CTX-M-266_ (Accession OR224962).

Amongst the 94 ST1788 genomes from Wales, 93 carried *bla*_SHV-232_ plus a *bla*_CTX-M_ variant, namely *bla*_CTX-M-15_ in 92 isolates and *bla*_CTX-266_ in SACU_33542. *bla*_OXA-1_ and *bla*_TEM-1_ were present in 89/94 (94.7 %) and 91/94 (96.8 %) genomes respectively. Alterations in the porins (*ompK35* and/or *ompK36*) were identified in 10/94 (10.6 %) genomes. A *bla*_OXA-48_ carbapenemase gene was identified in 4/94 (4.2 %) ST1788 genomes: three from patient 173 and one from patient 409; the distribution of *bla*_OXA-48_-carrying isolates in the phylogeny suggests that the gene was acquired on two separate occasions and there was no direct strain transmission between the two patients. The aminoglycoside acetyl transferase gene *aac(3’)-lla* was found in 87/94 (92.5 %) isolates, while the *aac(6’)-lb-cr* gene mediating resistance to aminoglycosides and fluoroquinolones was found in 89/94 (94.7 %) isolates; 86/94(91.5 %) isolates simultaneously carried both resistance markers. The streptomycin resistance genes *strA* and *strB* were simultaneously found in 91/94 (96.8 %) isolates. The majority of isolates (86/94; 91.5 %) carried the *qnrB1* fluoroquinolone resistance gene, while a mutated *gyrA* gene was found only in one isolate (1.1 %). All isolates carried the trimethoprim resistance marker *dfrA14* and 91/94 (96.8 %) isolates carried the sulphonamide resistance marker *sul2*. The distribution of AMR markers is detailed in Fig. 1 and Table S1.

### OXA-48 plasmid comparison

A hybrid assembly previously obtained for one of the four *bla*_OXA-48_-carrying ST1788 isolates (SACU_33542) demonstrated that the carbapenemase gene is present on a 63 499 bp IncL plasmid belonging to the pOXA-48-like family. Further analysis using the short-read data also suggested carriage of *bla*_OXA-48_ on the same plasmid type in the other three isolates (SACU_33476, SACU_33405 and SACU_37729) as all possess the IncL replicon and have mapping across ≥97.5 % of the pOXA48a reference plasmid.

## Virulence factors

A yersiniabactin locus was identified in 85/94 (90.4 %) ST1788 isolates from Wales; 84/85 (98.8 %) carried the *ybt16* variant on ICE*Kp*12, while the remaining isolates harboured *ybt10* on ICE*Kp*4. The distribution of *ybt16* variants in the phylogeny suggests that this was possessed by the ancestor of the ST1788 strains isolated in Wales. The KL2 capsule locus was identified in all isolates (including the three from Nigeria). Aerobactins, salmochelins, *rmpA* and *rmpA2* were not identified in any of the 94 strains from Wales included in this study. The distribution of virulence factors is detailed in Fig. 1 and Table S1.

## ST1788 PCR specificity

The use of *yphG* as a *K. pneumoniae *-specific marker was investigated. *In silico* PCR analysis of previously published oligonucleotides detecting *yphG* against the EuSCAPE collection of *K. pneumoniae* species complex genomes gave a positive result in 1623/1649 (98.4 %) *K *. *pneumoniae* genomes, while PCR detection was negative in the remaining 48 *K *. *variicola *, 17 *K *. *quasipneumoniae* or three *K*. *quasivariicola* genomes. *In silico* PCR detection of *yphG* was also confirmed in all 94 *K *. *pneumoniae* ST1788 genomes from Wales.

Pan-genome analysis using the above-mentioned public collection, together with a representative ST1788 genome (ARGID_35371) from Wales, initially identified four putative ST1788-specific genes (01404, 04677, 04687 and 04688) named with the progressive number as they appeared on the relevant assembly; larger genome collections were then screened with BLASTn to confirm their suitability as ST1788-specific markers. The additional genomes included 16 086 *K *. *pneumoniae* available in Pathogenwatch at the time of our study [28], the total sequenced collection of *Klebsiella* genomes from Wales (*n*=591) including 94 ST1788s and the NCBI-nr database. One gene (01404) was ruled out due to its presence in multiple copies in some genomes, as well as another two genes (04687 and 04688) that were present in five *K. pneumoniae* ST151 genomes from Pathogenwatch (query gene cover ≥75 %, nucleotide identity ≥99.5 %). The remaining gene (04677), encoding a reverse transcriptase and carried on an integrative conjugative element (ICE), was confirmed to be present only in ST1788 genomes including the three from Nigeria.

### *In vitro* analysis

#### AST results of ST1788 collection

Antimicrobial susceptibility testing (AST) of the 94 ST1788 isolates (Table S1) showed resistance to penicillins (both with and without β-lactamase inhibitor) with only one isolate (SACU_35951) susceptible to piperacillin/tazobactam, although with an elevated MIC of 4 mg l^−1^ close the breakpoint (8 mg l^−1^); 93/94 (98.9 %) isolates were simultaneously resistant to the third generation cephalosporins ceftazidime and cefotaxime; 89/94 (94.7 %) were resistant to gentamicin, 86/94 (91.5 %) to ciprofloxacin and 91/94 (96.8 %) to trimethoprim/sulfamethoxazole. Amikacin showed more potential activity, with only 11/94 (11.7 %) isolates tested resistant, but nevertheless 9/83 (10.8 %) susceptible isolates required testing with a gradient strip as the inhibition zone around the amikacin disc was close to the breakpoint of 18 mm; MIC results ranged between 6 and 8 mg l^−1^, therefore immediately below or equal to the breakpoint of 8 mg l^−1^. Performance of carbapenems was poorer for ertapenem with 11/94 (11.7 %) resistant isolates compared to imipenem and meropenem with 1/94 (1.1 %) resistant isolate each.

A specific multi-drug rsesiance (MDR) pattern was identified in 82/94 (87.2 %) isolates showing simultaneous resistance to penicillins, third-generation cephalosporins, gentamicin, ciprofloxacin and trimethoprim/sulfamethoxazole; three of these MDR isolates, namely SACU_35371, SACU_35765 and SACU_36406, were randomly selected to determine the level of resistance to cefotaxime, ceftazidime, gentamicin and ciprofloxacin to guide formulation of the in-house MHB to be used as part of the enhanced screening protocol. MIC results obtained by gradient strips (Table S1) indicated a high level of resistance to the first three antibiotics, while the ciprofloxacin MICs were just above the breakpoint, and therefore this antibiotic was not included in the broth. The collection of 94 ST1788 isolates was then used to validate the MHB: 88 isolates were able to grow, five did not grow due to susceptibility to gentamicin (MIC=0.5 or 0.75 mg l^−1^) and a further isolate did not grow due to susceptibility to cefotaxime (MIC=0.19 mg l^−1^) and ceftazidime (MIC=0.5 mg l^−1^).

#### PCR results on strain collection

Initial amplification of the *gfp* internal control was not successful. Pairwise alignment revealed possible interactions between the previously published dual priming *yphG* oligonucleotides and the *gfp* oligonucleotides. After removal of the polydeoxyinosine bridge and the 3′ end portion from the *yphG* forward and reverse primers, consistent amplification of the internal process control was observed in all reactions with no inhibition; no detrimental effect was noted on *yphG* amplification using the modified primers. The 04677 target was only detected from the 94 *K *. *pneumoniae* ST1788 strains. All but two *K. pneumoniae* strains, namely SACU_14501 (ST1393) and SACU_26589 (ST231), were positive for the *yphG* target. A strain of *K. grimontii* (SACU_32948) was also positive for *yphG*; BLASTn analysis showed that this strain carries a *yphG* variant with ca. 87 % nucleotide identity to that found in *K. pneumoniae *. All the remaining strains were PCR negative for the two targets.

#### Performance of screening protocols and PCR results

ESBL-producing *K. pneumoniae* were isolated using the standard screening method from 24/527 (4.5 %) screening samples collected from 10 patients (Table S3). Two different colony types, one susceptible to gentamicin and the other one resistant, were recovered from screening sample n. 414, making a total of 25 ESBL-producing *K. pneumoniae* isolates available. Nineteen isolates were characterized as ST1788, three as ST307, and the remaining three as ST513, ST834 and ST870. The two isolates from sample n. 414 were respectively characterized as ST1788 and ST307. The real-time PCR assay detected the ST1788-specific marker in all 19 ST1788 isolates, while *yphG* only was detected from the remaining six isolates.

A total of 96/527 (18.2 %) in-house MHBs used for the enhanced screening method showed visible growth after 24 h of incubation and a further 122/527 (23.1 %) after 48 h (Table S3). After sub-culture on ESBL selective medium, *K. pneumoniae* was isolated from 22/218 (10.1 %) MHBs with visible growth: 17 isolates were characterized as ST1788, three as ST307 and the remaining two respectively as ST147 and ST542. A further six strains were isolated after sub-culture on CLED only: two were characterized as ST870 and the remaining four respectively as ST14, ST23, ST147 and ST834. Real-time PCR detected ST1788-specific markers in all the 17 broths from where *K. pneumoniae* ST1788 was later isolated; two more MHBs inoculated with sample n. 37 and n. 142 collected from patients known to be colonized with *K. pneumoniae* ST1788 (patient 415 and 439) were also positive for the ST1788 marker (Ct=22.63 and 31.98 respectively), but no growth of *K. pneumoniae* was then obtained when the MHBs were sub-cultured on solid medium; *K. pneumoniae* ST1788 had been previously obtained by standard screening from sample n. 37, while neither standard or enhanced screening managed to grow it from sample n. 142. The *K. pneumoniae *-specific marker, *yphG*, was instead detected from a greater number of MHBs with visible growth, 55/218 (25.2 %) in total, but the distribution of Ct values was lower in those where *K. pneumoniae* then grew on ESBL selective agar (19.86≤Ct≤25.85) than in those where *K. pneumoniae* was not isolated (23.34≤Ct≤38.14) or was isolated only on CLED (29.92≤Ct≤35.58). Despite growth of *K. pneumoniae* ST542 from MHB n. 19, the *K. pneumoniae *-specific marker was not detected from the broth; WGS analysis and real-time PCR later confirmed that this isolate (SACU_37119) does not carry *yphG*.

Overall*, K. pneumoniae* ST1788 was isolated by both standard and enhanced screening from 14 samples collected from six patients (195, 327, 339, 418, 438 and 440). Standard screening isolated ST1788 from a further five samples from two patients (327 and 415) while enhanced screening isolated a further three samples from three different patients (339, 415 and 418). Taking into account the combined results, *K. pneumoniae* ST1788 was isolated from a total of 22 samples collected from seven patients (195, 327, 339, 415, 418, 438 and 440). One sample from a further patient (439) was only positive by real-time PCR tested on the in-house MHB (Table S4).

#### VNTR analysis and capsular typing by PCR

In total, 59/94 (62.7 %) *K *. *pneumoniae* ST1788 isolates were also typed using VNTR: 53/59 (89.8 %) isolates generated the 3,4,2,4,-,2,1,3,3,1,3 profile and 6/59 (10.2 %) isolates the 3,4,2,4,-,2,1,2,3,1,3 differing in just one locus (Table S1). The six isolates showing the less common VNTR profile were all obtained from the same ward of Hospital B (data not shown) or from a patient (n. 438) at Hospital C with prior admission at that particular ward of Hospital B. PCR analysis detected markers for the K2 capsular type in all the 59 isolates tested; however, the capsule up-regulation genes *rmpA* and *rmpA2* were not detected.

## Discussion

We present here the characterization of 94 *K *. *pneumoniae* ST1788 strains isolated in Wales which is so far the largest collection of this lineage to have been analysed. A small number of previous studies reported ST1788 elsewhere [25–27], and a previous study of ours aimed at characterizing the *Klebsiella* strains isolated from Wales included 43 *K *. *pneumoniae* ST1788 isolates, although an in-depth analysis of the lineage was not provided [24]. A significant proportion of ST1788 isolates in this study was obtained from one healthcare facility, Hospital B, in South East Wales. Both within- and between-hospital spread was demonstrated, suggesting a major role for nosocomial transmission of ST1788 within Wales.

MDR *K. pneumoniae* strains, especially those producing ESBLs and/or carbapenemases, have recently emerged as a major cause of healthcare-associated infections; concomitantly, hypervirulent *K. pneumoniae* strains expressing a defined set of acquired virulence factors are able to cause severe community-acquired infections [16, 22]. The majority of isolates collected during this study (78/94; 82.3 %) simultaneously carry *bla*_SHV-232_, *bla*_CTX-M-15_, *bla*_OXA-1_, *aac(3’)-lla*, *aac(6’)-lb-cr*, *qnrB1*, *dfrA14* and *sul2* resistance markers, the KL2 capsule locus, and the virulence factor yersiniabactin; 2/78 (2.6 %) of the above isolates also carried the *bla*_OXA-48_ carbapenemase. The resulting MDR phenotype, combined with the presence of yersiniabactins, make this lineage fit to survive and spread in both nosocomial and community settings; the ability to cause invasive infections was also documented, but the absence of further known virulence factors such as aerobactins, salmochelins, *rmpA* and *rmpA2* indicates a limited potential to cause severe disease in healthy individuals.

Fifteen *K. pneumoniae* ST1788 isolates grew from clinical samples collected from patients attending 11 different General Practices; all of these isolates were referred to SACU for investigation of possible carbapenemase production as suggested by local testing (data not shown). *K. pneumoniae* ST1788 strains isolated in similar circumstances that did not raise suspicion of carbapenemase presence would not have been referred to SACU for further examination and therefore not submitted for WGS analysis. This observation suggests that the transmission of *K. pneumoniae* ST1788 in the community in Wales has therefore occurred mostly unchecked and is likely to be much greater than that reported here. A similar issue probably affects the healthcare environment, but the introduction of the ESBL screening programme at Hospitals B and Hospital C together with referral to SACU of blood culture isolates for surveillance purposes from around Wales allowed us to capture *K. pneumoniae* ST1788 strains that would have otherwise been missed.

The MDR nature of the majority of ST1788 isolates is undoubtedly the major contributor to the nosocomial survival and spread of this lineage. In particular, ESBL production and consequent resistance to third-generation cephalosporins, such as ceftazidime and cefotaxime, is widely recognized as a marker of a potentially successful nosocomial pathogen [47]. The presence of a further β-lactam resistance marker, *bla*_OXA-1_, is another key contributing factor as it has been associated with increased MICs to penicillins/β-lactam inhibitor combinations such as amoxicillin/clavulanate and piperacillin/tazobactam [48]. Remarkably, only one out of the 94 isolates was susceptible to third-generation cephalosporins and another to piperacillin/tazobactam. The carbapenemase *bla*_OXA-48_ was identified in 4/94 (4.2 %) isolates from two patients with no evidence of direct transmission; resistance to the three main carbapenems, namely ertapenem, imipenem and meropenem, was observed only in one isolate, while the other three isolates were resistant only to ertapenem. *bla*_OXA-48_ is known to often cause low-level resistance to carbapenems [49].

At least one gene amongst *strA*, *strB*, *aac(3’)-lla* and *aac(6’)-lb-cr* with activity against aminoglycosides was identified in all 94 *K *. *pneumoniae* ST1788 strains. While there is scarce evidence of *strA* and *strB* activity against amikacin and gentamicin, the presence of *aac(3’)-lla* and *aac(6’)-lb-cr* has been widely associated with aminoglycoside resistance and is consistent with the high proportion (88/94; 94.7 %) of gentamicin-resistant strains in this study [50]. In the five isolates that were gentamicin-susceptible, either one or both *aac* markers was not present. Amikacin resistance was found in 11/94 (11.7 %) isolates and testing of a small number of susceptible isolates using gradient strips resulted in MICs (6 or 8 mg l^−1^) at the higher end of the MIC distribution (0.25–8 mg l^−1^) seen in the wild-type population (devoid of resistance markers) of *K. pneumoniae* isolates.

Simultaneous carriage of *bla*_CTX-M-15_, *bla*_OXA-1_ and *aac(6')-Ib-cr* has been identified in lineages, such as *E. coli* ST131, that are particularly successful as nosocomial pathogens; it has been suggested that detection or suspicion of their simultaneous presence in critically ill patients should be an indication for the use of or the switch to carbapenems [48].

Beside activity against aminoglycosides, *aac(6’)-lb-cr* has been demonstrated to be also active against fluoroquinolones, such as ciprofloxacin [51]; its simultaneous presence with *qnrB1*, a marker able to confer low-level resistance to fluoroquinolone by protecting the DNA gyrase [52], was identified in 85/86 (98.8 %) of the ciprofloxacin-resistant isolates in this study. The remaining ciprofloxacin-resistant isolate (SACU_36310, MIC=1.5 mg l^−1^) was carrying *aac(6’)-lb-cr* only, but this marker was also observed in three of the eight susceptible isolates (SACU_33328, SACU_36222 and SACU_36223); this observation is consistent with the statement reported by the EUCAST Subcommittee on WGS and Phenotypic AST that prediction of antimicrobial susceptibility from WGS data is presently not sufficiently accurate [53].

Finally, the presence of *dfrA14* and *sul2* resistance markers further limits therapeutic options. These genes are acquired via horizontal gene transfer and code for enzyme variants involved in folate biosynthesis, namely di-hydrofolate reductase and di-hydropteroate synthase, that are not inhibited by the widebroad-spectrum antibiotics trimethoprim and sulfamethoxazole which are often used in combination [54, 55].

Methodologies for isolation of microorganisms carrying specific resistance markers, such as ESBLs or carbapenemases, mainly rely on the direct culture of screening samples on selective agar media that are commercially available. Whilst these techniques have great utility within clinical microbiology laboratories, they lack the ability to select specific clonal types; therefore, an in-house enhanced screening programme targeting the *K. pneumoniae* ST1788 AMR pattern was implemented during this study. This approach allowed the isolation of a further three ST1788 strains from three different patients (namely 339, 415 and 418) already known to be colonized. The combined use of standard and enhanced methods increased sensitivity of the screening and showed the potential added value of targeting specific strains using liquid medium containing specific antibiotics, in this case ceftazidime, cefotaxime and gentamicin. A considerable number of broths were contaminated by growth of yeast cells present in the screening samples (data not shown); in future, addition of an antifungal to the relevant mix of antibiotics should be considered to decrease the number of broths needing sub-culture on solid media and PCR analysis. During the study period, *K. pneumoniae* ST1788 was isolated for the first time from a new patient (n. 440, screening sample n. 526) at Hospital B: both standard and enhanced screening methods yielded a positive culture and the isolate (SACU_37863) was included in the collection analysed in this study.

The multiplex real-time PCR was both effective and reliable in detecting the *K. pneumoniae* ST1788-specific marker from both isolates and broth-enriched screening samples. The assay allowed preliminary typing results to be more rapidly available to infection control colleagues during at a time when sequencing services were prioritizing work related to the SARS-CoV-2 pandemic. When both standard and enhanced screening methods failed to isolate ST1788 from a screening sample (n. 137) collected from a patient known to be colonized with ST1788, the real-time PCR assay instead yielded an unequivocal positive result. Strain-specific real-time PCR assays had already been designed and used in the past for at least two other pathogens, namely epidemic clones of *Pseudomonas aeruginosa* isolated from cystic fibrosis patients [56] and *Legionella pneumophila* clones that are common causes of Legionnaires’ disease, such as ST1 and ST47 [57, 58]. Specificity issues were observed for all the above-mentioned assays and in some cases, including the one presented here, target genes are present on mobile elements. Strain-specific PCRs should only provide preliminary typing results to be later confirmed by additional and more reliable typing methods.

A comprehensive analysis both *in silico* and *in vitro* was also performed on the assay detecting *yphG*, the *K. pneumoniae *-specific target. Although not present in all *K. pneumoniae* strains, *yphG* was identified in 98.4 % of the analysed genomes nd thus it can be considered a reliable marker. During the *in vitro* analysis, *yphG* was also detected from a strain of *K. grimontii* (SACU_32948) carrying a *yphG* variant with ca. 87 % identity to that found in *K. pneumoniae *; as only one *K. grimontii* strain was available for this study, it is not possible to state whether the above false positive result would be a major specificity issue.

In conclusion, despite international data showing *K. pneumoniae* ST1788 as being extremely rare worldwide, an MDR lineage of this strain has spread across both healthcare facilities and the community in South and West Wales. This lineage carries a plethora of AMR markers plus some virulence markers facilitating transmission and survival. The multiplex PCR assay described here allows unequivocal detection and preliminary characterization of this lineage; its implementation can aid rapid introduction of infection control measures.

## Supplementary Data

Supplementary material 1

Supplementary material 2

Supplementary material 3

Supplementary material 4

Supplementary material 5

Supplementary material 6
