# Supplementary material for: Clonal expansion and rapid characterization of Klebsiella pneumoniae ST1788, an otherwise uncommon strain spreading in Wales, UK
Source: Microb Genom. 2023 Sep 5;9(9):001104. doi: 10.1099/mgen.0.001104 (PMC10569728; doi:10.1099/mgen.0.001104)
Supplement: Supplementary material 1 [file mgen-9-1104-s001.pdf]

Supplementary Information

Clonal expansion and rapid characterisation of *Klebsiella pneumoniae* ST1788, an otherwise uncommon strain spreading in Wales, United Kingdom.

Massimo Mentasti[1], Sophia David[2], Jane Turton[3], Mari Morgan[4], Luke Turner[5], Joseph Westlake[1], Jonathan Jenkins[6], Catie Williams[6], Sara Rey[6], Joanne Watkins[6], Victoria Daniel[7], Shanine Mitchell[7], Gavin Forbes[7], Mandy Wootton[1] & Lim Jones[1]

Supplementary Table 1. List of *Klebsiella pneumoniae* ST1788 strains included in this study detailing full results of both phenotypic and genotypic analyses.
[Provided in Excel spreadsheet]

Supplementary Table 2. List of strains other than *Klebsiella pneumoniae* ST1788 included in this study detailing results of ST1788-specific real time PCR.
[Provided in Excel spreadsheet]

Supplementary Table 3. List of screening samples included in this study detailing results of standard and enhanced screening procedures, ST1788 PCR and WGS.
[Provided in Excel spreadsheet]

Supplementary Table 4. Summary of results obtained from screening sample that tested positive for ST1788.
[Provided in Excel spreadsheet]

Supplementary Table 5. Pairwise SNP differences amongst the 97 *K. pneumoniae* ST1788 isolates (post removal of recombined regions).
[Provided in Excel spreadsheet]

Microreact link: https://microreact.org/project/kp-st1788-phw

---

Supplementary data: nucleotide sequence of the real time PCR targets with highlighted PCR fragments (refer to following pages).

>04677 Reverse transcriptase (RNA-dependent DNA polymerase)

ATGGATATCGATTTAGATGATGTAAAAAAAGCATATCTTTACCTTAAGAGCTATGCCTACCATGAAAATCTGAATTTATTTTT
AAAGCAAAGAATTGCTGAATTTGAATCGGAAGGACAAGCTGATCATTTTATAGAAATTGTTAATGTTATTAAAGATGGAAATA
TCAAAAATAATGAAAACCTTATAAGATGGTTTGGTAAAGTTAACTTCCATGTTTTACCTAAAAGTATAGATATAAAAGGTGAG
TTAGAACAAAACGAACATAATAAAAGAAATGGTCTATTTATAAGCAATGTCCGGGATGCAGATATATACCAAATATCCAAAGT
GAATTATTTTATAAATGCTCCGGTACAGTTACATATAATTGATATGCTATGGTGCGTTTTTGCTGCTCCTGCAATTGAAACGT
TATTAGAAGATAATAGTTACGGTAATCGCATGCATCCCTCTGCTATTCGCTATGCGGTAGCAAAAC**GAACTAGCGGTGGTCAG**
GAAATATTTAAGAGATATATTGATCAATATAATTTATGGCGTGATCAGGCTGTTAGTATTGCTACAGATTTATCGAAAGAAGA
ACATGACGTTGCGCTATTGTCACTTGACCTAAAGTCATATTTTTATTTTGTTGATGTGGATTTCGATAAAATAAAAGAAGCTA
TAAATAGATATTACTCTCAGAGTGATACATTGCGAGATTTATCTATTCGGCTCACTGATATAATTGACGATTTATATTCTTTA
TATAATAAAATCACAAACACACTACAGAAAAAAACACATCCAAACTGCATAAATAAAAAGAGGTTACCAATAGGTTTAGCATC
ATCATCAATAATCGCGAACTGGTATTTAAGTGATTTTGATAAATCTATCGCGGATGAGGTTCGGCCTGCATATTATGGGCGTT
ATGTTGATGATATCTTAATGGTATTTAAAAGACCTAAATTAGATATGGAGAGGCCAATTGATTCTTTTGTTGATAATTATTTG
GCTGAAGTATTACTGAGAGATACTTCCGATGGTAATTATGCCATATCTGTAAATAATAACCTCTTGCCAGTACAAAAAGATAA
ACTCATATTGCAATTTTTTGATAAAGCTCATAGTCGAGCTGGGCTTGAAGTTTTAAACAAGAACTTGATGAGCGTAGCAGTG
CATTTAAGTTTTTACCAAGTGAACACATAGAAAAAGAACTGGATAAATTCGCCTACGATGTTCTTTATGATGGTTCTGCAAAC
AAATTACGTAGTATTGTTGGTCTTGCCGAAAATGAGACTGAGCTTGCTAAATATCTTACGAGTCATATTACTGCTCATAGACT
TTGTAAGTTAGATAAAAATAGCTCTGTTTTACCACAGTTAAAACAATTTTTTAAAGGGCAGAATGCATTAAGATTTCATAGGT
TGTGGGAGAAAATTTATCAATATTCTTTAGTTACGAAAAATTATTCATTTGCCACATCTTTTCATCACTATGTAATTAGAGAG
GTCAGTAAAGTAGAAGGTGCTCATACAGATAAAAATATAAAATTACGAACTTTGTCAATGAAGTTGATACGAGACCTTAAATA
TTACAATGAATTATCTTTGGCGATTACTGTCGGATTGCTTGATTTGAAGAGTCCAGTCAGTGAAAATGAATCACTCTTAATCG
GTAATGCTTTCTTAACTAATAGAACAAAGTTATCAGAACTGGTTTACTTTGGCTCTGATTTGCATGAATATTCATGGCAATTC
CGTTGCTCAAATTTAATTCGGCATCATTTAGTTGCATGGCCATTGCTAAATTATTCTAATTTCAATGGCGATCTTAGCAGTGA
AAATGGATTTATGAGCGGCGCTGGTTTTTCTCTTGAACAAGAAAAAATAAATTTTTCCCCGCGGTTTATTCATTATGACGAGT
GGGCTTTATTTAAACTTAATGGTTATTTATCAGATGGAAAAATTCTAAATGATTGGTTTTGTGATTCAATAGAGGAATATAGA

AATTATTTCTTTGATCAGGATTTCCCTGTTGTCGTGAAAAAAAATGAATGTACTACCAACCGGATTTTAAAGTCTGAAATACA
TATTGGTAACAAACAACCATGTAAGAATGTTAAATTAGCCGTTGCTAATATAGTTGTTGATGAAAGTGATATTTACTCCGCAA
TTCGTAAAGATACAAAGCCAAATCTTAGTTTTCCTCGTCAGGAAAAACTTTTTAATATTCTGAATTCTGCTCTTATTGAAAAG
GCAGATATGTTAGTTATGCCGGAGGTCGCTATACCTGTGAGCTGGCTGCCATTTATGGTTTCATTCTCAAGAAGACACCAGAT
TGGGTTGGTGTTTGGGTTAGAACATTGGGTTGTTGGAAATAGGGCATATAATGTTATTATAGAAGCATTACCATTCAAAATAT
CTGATAAATACAAATCCTGTGCAGTTACAGCGAGGTTAAAAAATCACTATGCGCCAGCAGAAAAGGATATGTTGGGGGATCTT
AGATTAACACCCGGTATTAGCTCCGATAATTTATATTACTATCATAGCGTTTCATGGCGTGGTGTTACTTTTGCAACATATAA
TTGCTTTGAATTATCAGATATAACTCATCGTGTTTTATTTAAATCAGAGATAGATTTGTTGTTTGCATGTGTTTGGAATAAGG
ACACGAATTACTATCAGCATATATTAGAGTCGGCAGTAAGGACCTTCACTGCTACACTGTGCAAGCTAATACATCACAATAT
GGAGGTTCGTGTATTCTTCGACCGACAAAAACAGAAAATAAAACAATGCTATATGTAAAAGGTGGAGAGAATTCTTGTGTGTT
GACCGCAAGTATTGATATATCAACCTTAAGAAGTTTCCAATTTAAATCCAAGCCAAATGTTAAAGATTCCTTTAAACATTTAC
CCCCAGGATATGATAGTGAAGCTGTATTAAAAAGGTAG

>yphG TPR repeat family protein DNA (Reverse strand)

ATGTTTCTGGAAAAACGTGTTTATCAAGGATCTTCCGGCAGCGTCTATCCCTACGGCGTGATTGATACCCTGACCGGCGAACG
CGAAATGCGCGATTATCAGGCAGTGTGGATGGAGAATGATTTCCTTCGGGTCATGCTGCTACCGGAGCTTGGCGGCCGTATTC
ACCGGGCATATGACAAAGTCAAACAACGTGATTTCGTCTATTACAATGAGGTGGTTAAGCCAGCCCTGGTAGGGCTGCTCGGG
CCCTGGATCTCCGGCGGAATCGAATTTAACTGGCCGCAACATCACCGGCCGACGACCTTTAAGCCGGTCGATTTTTCTATTCA
GCAGGGAGAAAACGGCGCGCAGACCGTATGGATGGGGGAAGCGGAGCCCATGCGCGGTTTGCAGGTTATGGCCGGGTTTACGC
TCTATCCTGACCGCGCGCTGATTGAAATCACTGGCAAGATCTTCAATGGTAACGCCACGCCGCGCCATTTCCTGTGGTGGGCC
AACCCTGCAGTAAAAGGCGGCGATGCCCACCAGAGTGTATTCCCACCCGATGTCACCGCTGTATTCGACCATGGTAAGCGTGA
TGTTTCCGCTTTCCCGATTGCCACCGGTACCTACTACAAAGTGGACTATTCTGCGGGGGTTGATATCTCCCGTTATAAAAACG
TCCCGGTACCCACTTCGTATATGGCGGAGAAGTCGGATTACGATTTTGTCGGCGCTTATCATCATGACGAGCGAGGCGGGTTA
CTGCATGTTGCCGATCACCATGTCTCTCCTGGTAAGAAACAGTGGAGCTGGGGGTACGGGGATTTTGGCCAGGCCTGGGATCG
AAATCTGACCGATGAAAATGGCCCTTATATTGAGCTGATGACCGGCGTCTTCACCGATAACCAGCCTGACTTTACGTGGCTTG

CGCCTTATGAAGAGAAGGTGTTTGTACAAAATTTCCTTCCCTACAGCGAGTTAGGGATGGTGCAAAATGCCAATACACAGCTG
GCACTGAAGCTGGTTCGCGAGTCTGGGCAACTGCTGCTTGGCGTATATGCCATTGCTCCGCTAAATCATATCGTGGTTGAACT
CAGCGCTGACCATCAGCCATTGTACGAAACTCAGCTGACGCTGAAGCCCGGTGAGAGCTGGCAGCAGACGCTGCCGGAAAATG
GCGTCGGGCGCTTAACCCTCAAAGTGAAAACCGCCGAGAACCAGCCGCTGCTGGATTATCAGGAACATATTACCCAACAGACG
CCGCTGCCCGAACCAGCTATAGCGCCCGCGTTACCGGAAGCGATCCACAATGGCGACGAGCTCTATTTTATCGGCCAGCACCT
TGAACAATACAACCACGCAAGCCGCTACGCCGGGGATTACTATCGTCGGGCGGTTGAGCTCGATCCGCAGGATTATCGCAATA
ATGTTGCCCTCGGTACCCTGGCGTTCAACAGTGCCGACTGGGCGCTGGCGGAACAGTGTGCCCGTGCTGCGCTACAGCGCGCG
CATCGTTTGAATAAGAACCCGCGAGATGGCGAAGCCAGTATGCTGTTTGCCAGCGTGCTGGAAAGGATGGGGGATGATGCAGG
CGCCTGGGATCACTATTACAAAGCCTCCTGGAGCGGTAACTGCCGTGACGCCGCCTGGTGGTCGCTGGCACGGCTGGCGATGA
AGCGCGGTGATGTTGCCGATGCGCTGGAAAAAGTAAACACCAGTCTGCGGTTCAACGCCAGCAATCCTCTGGCGATGGGGTTG
AAAGCGCTGGCATTAGCCAACAGTGGGCAGAAGAAAGCAGCGCTGGAGTTTATTTTCGCCTCTCTTGAGCAATACCCATTGAG
CTATCCGCTGCACTGCGCGCGCTGGATGATTGAGCGGAGCGACGACGCGCGAGAAACCCTGCTGCGCATCACGGGGCGCAGAG
GGGTCAACGCCAGCCTGCTGGCGGGTTGGCTGC**TCTCTATCGGACAGACGTCGGCGGTGAAAGAGGTTCTTGCGGTTCTGGAC**
AGTCAGGAAGCGCTGCCGATGCTCTGGCGTGCGTCGCTAAGTGATGATGCCAATGAACGCCAACAGTTTATTGCCGCCGCCGA
GCACTGCCATGCGCACAATGTTCGTTTCCCTAACTCGCTCGATGAAGTTCAGATGCTGCAGTCGCTGGGCGACAGCGCTTTTG
CCCGCTATCTACTCGGTTGCTTCTGGTACAGCAAACGTCGCTATGACGAGGCGGTGAGCTGCTGGCGCGAGACGCTCGAAAAA
TCTCCCGATTATGCGCCCGCTCATCGTCTGCTGGGCGTTTACTCCTGGAATAAACAGCAGGATGCAACGCAGGCGCTGGCTTA
TCTGCAGCGCGCCGTAGCGCTGGAGCCGGACAATGCGCGCTTCCTGTTTGAACTCGATTTTTTGCAAAAATTACTGGCCAGGC
CGGTACATGAGCGACTGACGACGCTGGTGGAACGTAAAGCCGTGGTGCTGAAGCGGGACGACCTGACCGCTGAGCTGCTAAGC
CTGTGGAACGCGTCCGGTCATTATGCCGATGCCGCCGCGATTCTGGATACGCGCGTATTCCATCCCTGGGAAGGAGGAGAAGG
GAAAATCACCGGCCAGTATTTGCTCAATCAGCTCCATCGGGCATTAGAGTTCATCGAGCGTGGGGCGTTTAAACAGGCCACGG
ACTGTCTGAAAGCCGCATTACGCTATCCCGACAATCTGGGGGAGGGGCGACTGCCTGGGCAAACGGATAACGATATCTGGTAT
TTGCTGGGCTACTGCGCAGAACAGGCTGGGGATGCGCAGCAGGCGGCGGAATATTACCAGCTTGCCCGGCAAGGGGGCTCAAC
GCTGGACGCCGGGCGGTACTACAACGATCAACCCGCCGATTACCTTTTCTGGCAGGGCATTGCGCTGCGTAAGAGCGGTAACC
CAGCGCAGGCCGAGCAGCACTTCCGCCATTTTATCGACTGGGCCGCGCAGCATCGGGATGACGTTCCGCAAGTCGATTTTTTT
GCCGTCTCTCTTCCGGACCTGGTGGTTCTCGATGTATCAGCGCAGCAGAGACATCAGCAGCACTGCTTGTTTATTGAAGCGCT

GGGCCATCTGGGGCTGGGCAACGTATCCGCCTGCCAGCAGAGGATGCAGCAGCTTCTGCAAATAAATCCGGCTCATGATAAGG
CGCATCTGATTCGCCATGCCCTGCAAAGCGGCATTTTTTCCTGA

>M62653.1 Aequorea victoria green-fluorescent protein DNA, complete cds

ATGAGTAAAGGAGAAGAACTTTTCACTGGAGTTGTCCCAATTCTTGTTGAATTAGATGGTGATGTTAATGGGCACAAATTTTC
TGTCAGTGGAGAGGGTGAAGGTGATGCAACATACGGAAAACTTACCCTTAAATTTATTTGCACTACTGGAAAACTACCTGTTC
CATGGCCAACACTTGTCACTACTTTCTCTTATGGTGTTCAATGCTTTTCAAGATACCCAGATCATATGAAACAGCATGACTTT
TTCAAGAGTGCCATGCCCGAAGGTTATGTACAGGAAAGAACTATATTTTTCAAAGATGACGGGAACTACAAGACACGTGCTGA
AGTCAAGTTTGAAGGTGATACCCTTGTTAATAGAATCGAGTTAAAAGGTATTGATTTTAAAGAAGATGGAAACATTCTTGGAC
ACAAATTGGAATACAACTATAACTCACACAATGTATACATCATGGCAGACAAACAAAAGAATGGAATCAAAGTTAACTTCAAA
ATTAGACACAACATTGAAGATGGAAGCGTTCAACTAGCAGACCATTATCAACAAAATACTCCAATTGGCGATGGC**CCTGTCCT**
TTTACCAGACAACCATTACCTGTCCACACAATCTGCCCTTTCGAAAGATCCCAACGAAAAGAGAGACCACATGGTCCTTCTTG
AGTTTGTAACAGCTGCTGGGATTACACATGGCATGGATGAACTATACAAATAA
